# Eukaryotic protein glycosylation: a primer for histochemists and cell biologists

**DOI:** 10.1007/s00418-016-1526-4

**Published:** 2016-12-23

**Authors:** Anthony Corfield

**Affiliations:** Mucin Research Group, School of Clinical Sciences, Bristol Royal Infirmary, University of Bristol, Bristol, BS2 8HW UK

**Keywords:** Eukaryocyte, Glycans, Glycoprotein, Glycosylation, Histochemistry, Mucin

## Abstract

Proteins undergo co- and posttranslational modifications, and their glycosylation is the most frequent and structurally variegated type. Histochemically, the detection of glycan presence has first been performed by stains. The availability of carbohydrate-specific tools (lectins, monoclonal antibodies) has revolutionized glycophenotyping, allowing monitoring of distinct structures. The different types of protein glycosylation in Eukaryotes are described. Following this educational survey, examples where known biological function is related to the glycan structures carried by proteins are given. In particular, mucins and their glycosylation patterns are considered as instructive proof-of-principle case. The tissue and cellular location of glycoprotein biosynthesis and metabolism is reviewed, with attention to new findings in goblet cells. Finally, protein glycosylation in disease is documented, with selected examples, where aberrant glycan expression impacts on normal function to let disease pathology become manifest. The histological applications adopted in these studies are emphasized throughout the text.

## Introduction

Histochemists and cell biologists are familiar with the ubiquitous presence of glycans. In view of the increasing awareness that their structure is an ideal platform to store information [Winterburn and Phelps 1972; Gabius 2009, 2015; please see also the introduction to this theme issue (Gabius and Roth 2017)], a survey of their characteristics is timely. In connection with the overview on glycolipids (Kopitz 2017, this issue), an introduction to protein glycosylation is provided here. Present in archae- and eubacteria and in Eukaryotes (Reuter and Gabius 1999; Patsos and Corfield 2009; Wilson et al. 2009; Zuber and Roth 2009; Corfield 2015; Corfield and Berry 2015; Tan et al. 2015), protein glycosylation is shared by organisms of all three urkingdoms, associated with diseases when aberrant (Hennet 2009; Hennet and Cabalzar 2015). Starting with structural aspects, functional implications are then exemplarily discussed.

## Glycosylation of proteins: general aspects

**Fig. 1:**
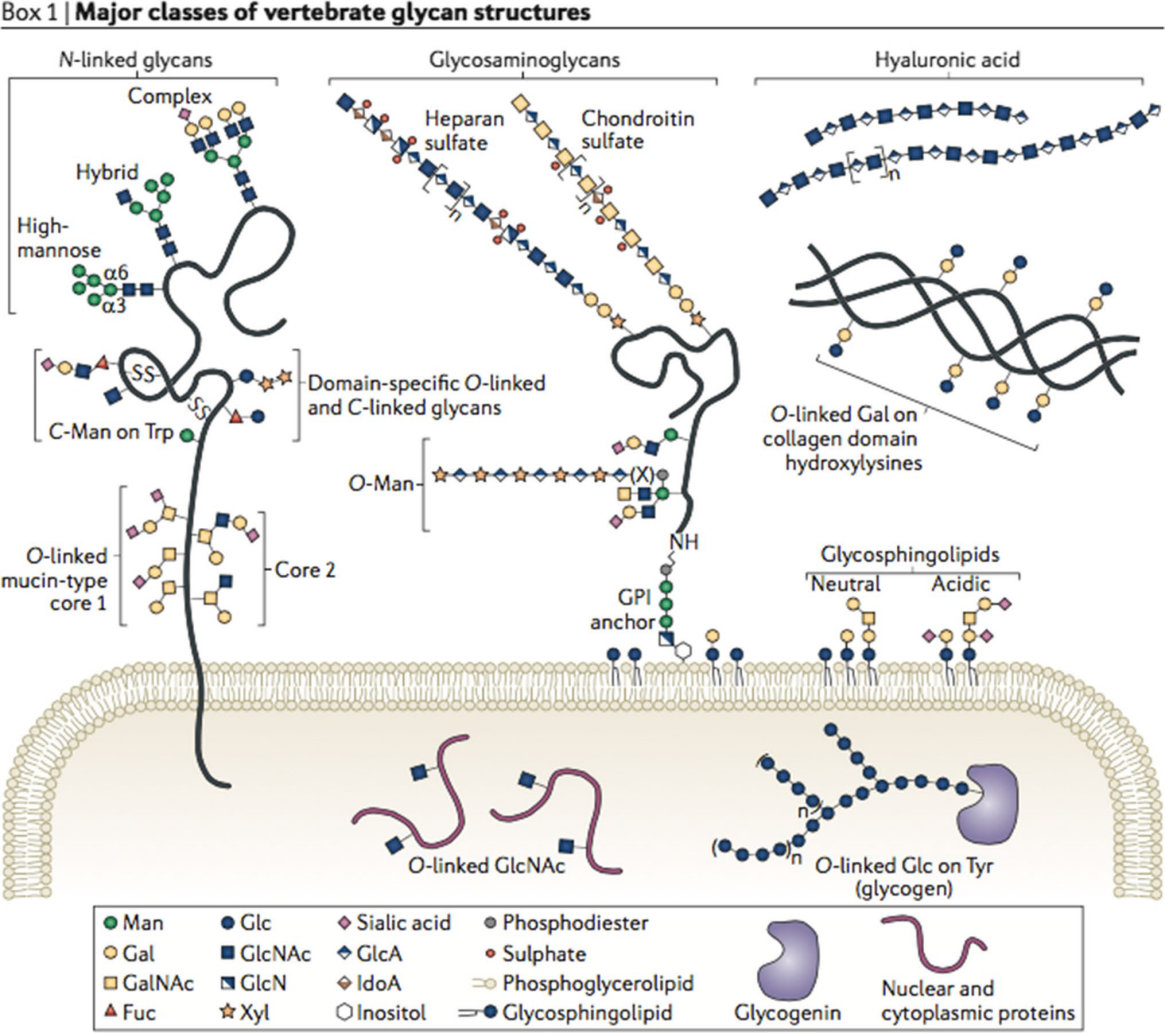
Classes of vertebrate glycan structures. Membrane and secreted proteins have *N*-glycan, Glc*N*Ac to asparagine as oligomannose, complex or hybrid forms, or *O*-glycans linked through Gal*N*Ac to serine/threonine with eight core structures and extension. Glycosaminoglycans have a core linkage tetrasaccharide to protein, with subsequent disaccharide repeats and characteristic sulphation patterns. They may be secreted, transmembrane or GPI-anchored. Hyaluronan is not linked to a protein. *O*-Mannosyl residues may be extended. *O*-Glucose and *O*-fucose are found in EGF domains of some proteins. C-Mannose is attached to protein tryptophan side chains. Single β-*O*-Glc*N*Ac is found on many cytosolic and nuclear proteins. The collagen disaccharide is linked to hydroxylysine and through galactose. Glycogen is linked through glucose unit to a tyrosine in glycogenin. Glycosphingolipids contain glycans linked to a ceramide carrier; from Moremen et al. (2012), with permission

Most of the proteins are subject to glycosylation by a wide variety of enzymatic mechanisms. The length of the conjugated glycan ranges from a single sugar moiety to branched structures and the long glycosaminoglycan chains (Fig. 1; for information on proteoglycans, please see Buddecke 2009).

**Fig. 2:**
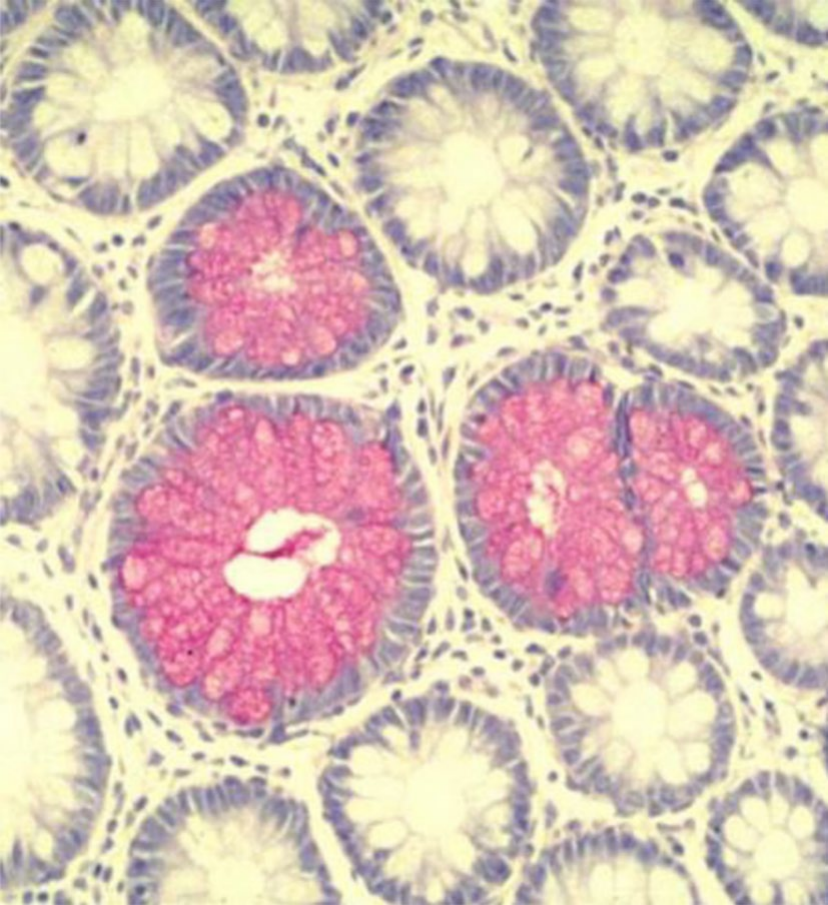
mPAS detection of sialic acids in human colon. Mucus stored in goblet cell thecae. Staining of the colonic mucosa with the mild periodic acid-Schiff reaction stains non-*O*-acetylated sialic acids and demonstrates the location of the mucus prior to secretion; from Corfield (2011), with permission
- Detection of glycan by probes with specificity to glycans, i.e. monoclonal antibodies (such as the CD-based reagents specific for the T/Tn antigens; for an overview, please see Gabius et al. 2015) or lectins (for an introduction to lectins and their application in cyto- and histochemistry, please see Kaltner et al. 2017; Manning et al. 2017, this issue). Working with cytological specimen or tissue sections, glycophenotyping is readily feasible with labelled lectins by various microscopical techniques (Roth 1993, 1996, 2011; Habermann et al. 2011). Using chemically prepared compounds as inhibitors (Murphy et al. 2013; Roy et al. 2016), structural and topological aspects of the specificity of lectin binding can be analysed (André et al. 2016; Roy et al. 2017, this issue). In addition to their application, lectins have found a broad range of applications for glycoprotein analysis (for compilation, please see Table 1 in Solís et al. 2015). These versatile assays also shape the notion that such interplay will have physiological relevance (for information on tissue lectins, please see Gabius et al. 2016; Kaltner et al. 2017; Manning et al. 2017; Mayer et al. 2017; Roth and Zuber 2017, this issue).

This wide spectrum of structural modes of glycosylation requires access to detailed information available on the presence of glycans. Representative techniques are listed as follows:

- Detection of glycans as carbohydrates in glycoproteins using chemical assays. This can be applied for screening in standard fractionation techniques such as high-performance liquid chromatography, size fractionation chromatography, ion-exchange chromatography, electrophoretic methods and density gradient centrifugation (Brockhausen et al. 1988; Nakagawa 2009; Marino et al. 2010).
- Detection of glycans as carbohydrates in tissue sections using chemical assays to provide morphological data regarding the localization of the carbohydrate/glycoprotein (Filipe and Branfoot 1983; Buk and Filipe 1986; Warren 1993; Filipe and Ramachandra 1995; Corfield and Warren 1996) (for an example on the identification of *O*-acetylated sialic acids in human colon using the mild-PAS method, please see Fig. 2).

## Glycosylation: biological roles

**Fig. 3:**
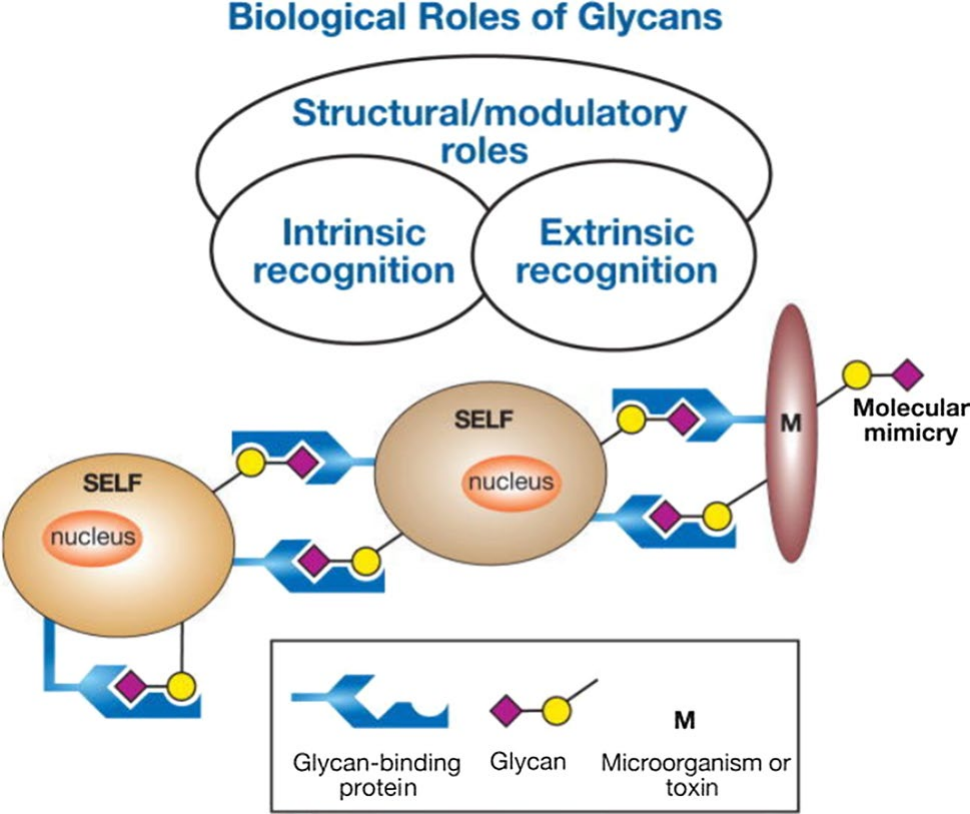
Biological roles of glycans. A general classification of the biological roles of glycans is presented, emphasizing the roles of organism proteins in the recognition of glycans; from Varki and Lowe (2009), with permission

Glycosylation is a flexible co- and posttranslational modification that has been adopted by Eukaryotes to create a dynamic strategy applicable in modern biology. As many options are possible, an overview of the biological relevance of glycan chains in glycoproteins is shown in Fig. 3.

Backed by exemplary references, special aspects are highlighted:

- Impact on the physicochemical properties of the glycoprotein molecule. The secreted mucins are an example, where viscoelasticity and gel formation establish a protective barrier on mucosal surfaces (Newton et al. 2000; Pearson et al. 2000; Atuma et al. 2001; Allen and Flemström 2005; Gustafsson et al. 2012; Johansson and Hansson 2012; Verdugo 2012; Berry et al. 2013; Birchenough et al. 2015).
- Docking sites for tissue lectins, hereby serving a broad range of functions including adhesion, growth regulation or routing (for further information, please see Gabius et al. 2011, 2016 and in this issue, Kaltner et al. 2017; Manning et al. 2017; Mayer et al. 2017; Roth and Zuber 2017). The quality control and the specific delivery of glycoproteins in tissues and cells are illustrative examples. Specific functions of individual glycoproteins are related to their location and selective expression. The glycans serve as postal code for routing and delivery, for example for asialoglycoproteins, lysosomal enzymes carrying mannose-6-phosphate or glycoproteins in galectin-dependent apical/axonal transport (Kornfeld et al. 1982; Stechly et al. 2009; Velasco et al. 2013; Higuero et al. 2017; Manning et al. 2017, this issue).

In order to illustrate the importance and scope of protein glycosylation it is necessary to enumerate the range glycan structures that have been identified and which are carried by glycoproteins. Table 1 gives an overview of the broad scope of glycan structures found in Eukaryotes. The main types of glycosylation are *N*-linked and *O*-linked glycans, with a considerably smaller group of C-linked glycans.

**Table 1:**
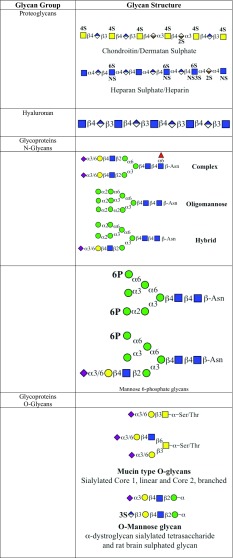

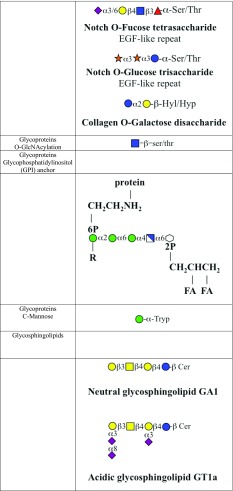
Main types of glycan structures

Major groups of eukaryotic glycans. Examples of the general types of glycan, largely drawn from animal examples, are shown. Key: yellow circles, d-galactose; yellow squares, *N*-acetyl-d-galactosamine; blue circles, d-glucose; blue squares, *N*-acetyl-d-glucosamine; blue/white squares, d-glucosamine; green circles, d-mannose; red triangles, l-fucose; purple diamonds, *N*-acetyl-d-neuraminic acid; light blue diamonds, *N*-glycolyl-d-neuraminic acid; blue/white diamonds d-glucuronic acid; orange/white diamonds, l-iduronic acid; orange stars, d-xylose; white diamonds, myo-inositol. All glycosidic linkages are shown as α or β, with the corresponding position; for example, β4, β1,4 linkage. *2S* 2-*O*-sulphate, *3S* 3-*O*-sulphate, *4S* 4-*O*-sulphate, *6S* 6-*O*-sulphate, *2P* 2-*O*-phosphate, *6P* 6-*O*-phosphate, *Asn* asparagine, *CH*_*2*_ *CH*_*2*_ *NH*_*2*_ ethanol amine, *FA* fatty acid, predominantly palmitate, *Hyd* hydroxylysine, *Hyp* hydroxyproline, *NS N*-sulphate, *Tryp* tryptophan, *R* various glycan substitutions occur at the initial mannose in GPI anchors; from Corfield and Berry (2015), with permission

*N*-Linked glycans are attached through an *N*-glycosidic bond between asparagine and β-*N*-acetyl-d-glucosamine (Glc*N*Ac). The asparagine residues are associated with the recognition sequence Asn-X-Ser/Thr. This sequence and the associated synthetic pathway are conserved in evolution for all of the metazoan (Aebi 2013; Breitling and Aebi 2013). The *N*-glycans contain a common, branched core comprising Manα1,6(Manα1,3)Manβ1,4Glc*N*Acβ1,4Glc*N*Acβ1-Asn-X-Ser/Thr and this is extended to yield three different types, oligomannose, complex and hybrid (Zuber and Roth 1990). Common features occur in the extension of the *N*-glycan core, generation of two antennae from the Manα1,6(Manα1,3)Manβ1,4Glc*N*Acβ1,4Glc*N*Acβ1Asn-X-Ser/Thr core. Second, the core is extended to yield oligomannose forms containing only mannose, formation of complex types having antennae terminated with a sialylated *N*-acetyllactosamine trisaccharide, plus a fucose on the internal Glc*N*Ac linked to the asparagine and finally hybrid types containing both oligomannose linked to Manα1,6 and complex units attached to the Manα1,3 residues (Aebi 2013; Breitling and Aebi 2013).

The process of *N*-glycosylation, starting co-translationally, is common across the Eukaryotes in accordance with their comprehensive range of biological functions. The enzymes responsible for the stepwise generation of the precursor glycan utilize a dolichol pyrophosphate lipid carrier and follow a series of trimming and processing steps that are conserved across the Eukaryotes. A series of three cytoplasmic glycosyltransferases, initially a Glc*N*Ac transferase followed by mannosyltransferases, result in the formation of the Man5Glc*N*Ac2 pentasaccharide. Subsequent extension occurs in the lumen of the endoplasmatic reticulum and the dolichol-oligosaccharide is translocated by a flippase. In the ER lumen a series of manipulations occur to generate the range of *N*-glycans required for the tissue (Zuber and Roth 2009; Aebi 2013; Breitling and Aebi 2013).

Oligosaccharyltransferase (OST) is the principal enzyme in the *N*-glycan pathway. It catalyses the transfer of the glycan from the dolichol phosphate-oligosaccharide to an asparagine in Asn-X-Ser/Thr motifs on acceptor polypeptides. OST is a hetero-oligomeric complex comprising 8 subunits in most Eukaryotes. The transfer reaction catalysed by OST is exclusive, showing strict substrate specificity applicable to wide range of protein acceptors (Zuber and Roth 2009; Aebi 2013; Breitling and Aebi 2013).

*N*-Glycosylation is closely linked with important glycoprotein regulatory events. Protein folding is mediated by the chaperones calnexin and calreticulin and ensures that glycoproteins that exit the ER are correctly folded (Roth 2002). Trimming of the terminal triglucosyl unit by α-glucosidases I and II is followed my monitoring of the glycoprotein. In the case that folding is incomplete a single α-glucose residue is transferred to the α1,2mannose unit on the α1,3mannosyl antenna. Recycling ensues and the glycoprotein is reassessed in the same manner. Those glycoproteins that do not fold properly are eliminated by ER-associated degradation (Roth 2002; Aebi 2013; Breitling and Aebi 2013; Roth and Zuber 2017).

The second most common type of glycosylation, the *O*-glycosidic linkage coupling serine or threonine to α-*N*-acetyl-d-galactosamine (Gal*N*Ac), also known as mucin-type glycosylation, as it is the major glycosylation found in this large group of heavily glycosylated proteins (Corfield 2015). Other non-mucin-type *O*-glycans have been detected, and these are described later. The *O*-glycans present in mucins are located in variable number tandem repeat domains, which vary in size and sequence between the different mucins (Hattrup and Gendler 2008; Thornton et al. 2008; Bafna et al. 2010; Kreda et al. 2012; Corfield 2015). *O*-Glycans do not have a peptide recognition sequon, as established for *N*-glycans, but are characterized by eight different core structures, as shown in Table 2. The most frequently observed are cores 1, 2, 3 and 4.

**Table 2:**
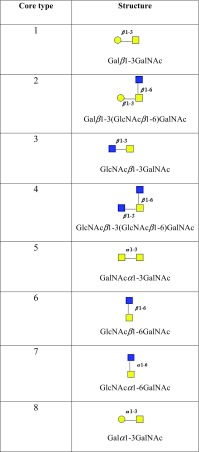
Mucin core structures

*O*-Glycan core structures found in eukaryotic mucins. Key: yellow circles, d-galactose; yellow squares, *N*-acetyl-d-galactosamine; blue squares, *N*-acetyl-d-glucosamine; all glycosidic linkages are shown as α or β; from Corfield (2015), with permission

The initial transfer of a Gal*N*Ac to serine and threonine residues in proteins is catalysed by a family of Gal*N*Ac transferases (Patsos and Corfield 2009; Tabak 2010; Gerken et al. 2011; Bennett et al. 2012; Gerken et al. 2013; Revoredo et al. 2016), the site of action localized immunohistochemically by electron microscopy (Roth et al. 1994). The core structures are extended through *N*-acetyllactosamine backbone repeat unit of type 1 (Galβ1,3Glc*N*Ac-) or type 2 (Galβ1,4Glc*N*Ac-) or the blood group antigens I (Galβ1,3GlcNAcβ1,3(GlcNAcβ1,6)Galβ1,4-) and I (Galβ1,4Glc*N*Acβ1,3Galβ1,4-R). Peripheral glycosylation of these structures is extensive and includes ABO and Lewis blood groups together with sialylated, fucosylated and sulphated glycans. The pathways responsible for the biosynthesis of these glycans are well studied (Schachter and Brockhausen 1992; Brockhausen and Schachter 1997; Patsos and Corfield 2009; Corfield 2015; Corfield and Berry 2015). Unique for mucin glycosylation is the α-Glc*N*Ac terminus of core 2 *O*-glycans in the gastrointestinal tract, which is readily detectable with the plant lectin GSA-II (Nakayama et al. 1999; André et al. 2016).

A large group of cytosolic and nuclear proteins, which carry multiple additions of a single β-*O*-Glc*N*Ac unit linked to serine and threonine hydroxyl residues, has been reported. The same serine and threonine residues are also sites for phosphorylation, prompting consideration of a mutual relationship between these two modifications (Butkinaree et al. 2010; Ma and Hart 2014). The cycling of β-*O*-Glc*N*Ac and phosphate has functional roles and is mediated by an *O*-Glc*N*Ac transferase (Zimmerman et al. 2000) and an *N*-acetyl-d-glucosaminidase (Zhu-Mauldin et al. 2012). *O*-Glc*N*acylation is common throughout the metazoans.

Further *O*-glycan families have been identified. *O*-Mannose α-linked to serine and threonine residues is commonly found in the metazoans, largely in skeletal muscle and the brain and nervous system (Lommel and Strahl 2009; Vester-Christensen et al. 2013; Panin and Wells 2014; Neubert and Strahl 2016). A tetrasaccharide, Neu5Acα2,3Galβ1,4Glc*N*Acβ1,2ManαSer/Thr is found in the skeletal muscle protein α-dystroglycan, and most *O*-mannose glycans are related to this structure despite additional modifications with fucose, glucuronic acid and sulphate (Panin and Wells 2014).

Proteins with epidermal growth factor domains carry glycans *O*-linked to peptide serine or threonine through α-fucose and β-glucose. Urokinase, factor XII, cripto factor IX, thrombospondin type 1 repeats, Notch, Delta and Serrate have been identified. The epidermal growth factor (EGF) domains of these proteins carry the *O*-fucosylated glycans of the type Neu5Acα2,3/6Galβ1,4Glc*N*Acβ1,3FucαSer/Thr, or smaller and a consensus sequence Cys^2^X_4–5_Ser/ThrCys^3^ has been identified (Takeuchi and Haltiwanger 2014). The most common *O*-glucosyl structure is (Xylα1,3Xylα1,3Glcβ-*O*-) and a peptide consensus domain Cys^1^XSerXProCys^2^ reported (Gebauer et al. 2008; Takeuchi et al. 2012). The disaccharide, Glcα1,2Galβ-, found in collagen, is well known. The posttranslational modifications of the peptide to create the hydroxylysine and hydroxyproline generate the sites for β-*O*-galactosylation to form Glcα1,2Galβ-*O*-Hyl/Hyp (Schegg et al. 2009).

A peculiar type of protein glycosylation, without a typical glycosidic bond, is formation of C–C linkages between α-mannose and the indole unit of tryptophan residues. The motif WXXW carries the glycans and is found in the Cys-D domains of several mucins, including the mucins MUC2, MUC5AC and MUC5B. The number of Cys domains varies between mucins, with 2 in MUC2, 7 in MUC5B and 9 in MUC5AC (Hofsteenge et al. 1994, 2001; Perez-Vilar et al. 2004; Ambort et al. 2011). Cys domains function in protein folding and mediate subcellular localization and trafficking in the endoplasmatic reticulum and Golgi membranes (Perez-Vilar et al. 2004; Ambort et al. 2011). C-Mannosylation in the mucin Cys domains governs the normal development and secretion of the mucins and when faulty induces ER stress, with mucins remaining in the ER (Desseyn 2009). Strengthening of the mucus layer in the gut lumen could be achieved by delivery of a tandem repeat molecule containing 12 repetitive Cys domains (Gouyer et al. 2015; Desseyn et al. 2016).

Many proteins possess a glycosylphosphatidylinositol (GPI) membrane anchor, attached to their carboxyl terminal. This ensures presentation of the protein on the external cellular surface where many important biological events occur (Ferguson et al. 2009; Shams-Eldin et al. 2009). As the anchor can be cleaved by phosphatidylinositol phospholipase C, release of the protein can be mediated by the cell and correlated with function at the site of expression. The basic core structure of the GPI anchor is ethanolamine-phosphate-6Manα1,2Manα1,6Manα1,4GlcNα1,6-*myo*-inositol-1-phosphate-lipid. Proteins are attached to the amino group of the ethanolamine through their C-terminal carboxyl groups. A number of variations on this core are found, of particular interest is the addition of a palmityl group to the C2 group of myo-inositol as this blocks the action of phosphatidylinositol phospholipase C and regulates the biological half-life of the GPI protein in the membrane. GPI anchors are common across all Eukaryotes (Ferguson et al. 2009; Shams-Eldin et al. 2009).

In order to generate the spectrum of glycan structures found on proteins, and indeed other glycan carriers such as lipids [for an introduction to glycolipids, please see Kopitz (2017) in this issue], individual cells must synthesize the glycans, with the required sequence. The metabolic pathways that are responsible for this process include the generation of a series of precursors from monosaccharides, the nucleotide sugars; sugar transporters that ensure that the necessary intermediates are available in the cell to generate the precursors; glycosyltransferases, which transfer the sugars to the acceptor protein to form the desired glycan structure, plus a number of other proteins which contribute to the formation of the final glycoprotein structure designed for specific biological function (Schachter and Brockhausen 1992; Liu et al. 2010; Corfield 2015); insights into details of branch-end elaborations, typically by sialylation, are presented by Bhide and Colley (2017, in this issue). These pathways are integrated to allow additional manipulation of the product glycoprotein. They also include catabolic manipulations, which may be linked to normal turnover and degradation, or specific modifications, which generate biologically active glycoforms and the salvage pathways feed back into the overall metabolism of glycoprotein metabolism. Further detailed information regarding glycobiology in this context can be found on the CAZy and Consortium for Functional Glycomics websites, see http://cazy.org and http://functionalglycomics.org.

It is clear that this is a complex system, with many options necessary to form required glycoproteins at specific cell sites. Much of this specificity is achieved through the selective expression of glycosyltransferases, such that the combination allows only certain structures to be formed. The omission of glycosyltransferases will preclude the formation of certain glycans. As the glycan sequence is generated on a non-template basis, in contrast to nucleic acids and proteins, this is the remaining metabolic option to achieve any kind of sequence specificity and is clearly open to error through metabolic fluctuation (Brockhausen 2003; Breitling and Aebi 2013; Takeuchi and Haltiwanger 2014; Corfield 2015; Corfield and Berry 2015; Neubert and Strahl 2016).

## Glycosylation in organisms

The global presence of protein glycosylation in the living world implies important biological function and development during evolution. It is to be expected that the structural features and physiological advantages will be carried forward, passed across species and provide biological markers in organisms. This section draws on examples of glycan occurrence and details the development relevant to the Eukaryotes. Several reviews contain a broad overview of these aspects with regard to the Eukaryotes and should be used as a supplement to this paper, e.g. Wilson (2002), Gerken et al. (2008), Dell et al. (2010), Lauc et al. (2014), Corfield and Berry (2015), Xu and Ng (2015). Much of the background to established glycosylation patterns in the Eukaryotes is in parallel with that reported for bacterial systems (Bäckhed et al. 2005; Moran et al. 2011; Tan et al. 2015) and it is certainly helpful to consider the bacterial systems, as they have a range of evolutionary aspects of interest.

The glycocalyx is a major characteristic of Eukaryote cells (see section “Glycosylation in cells”). It is this surface location where major interactions between cells takes place and enables communication and recognition processes to take place. Knowledge of the structural features is essential if the communication and functional elements of Eukaryote physiology are to be understood. They enable design of experimentation to reveal the precise nature of these interactions and provide a basis for the preparation of analogues and inhibitors to dissect the biological pathways involved.

As indicated in “Glycosylation of proteins” section, the chemical nature of glycans lends itself to the construction of structures with considerable variety and therefore excellent possibilities to adopt a system, which codes for functional roles in biology. It is significant that although a wide range of sugars are available in biological environments the glycan structures found in nature is limited, suggesting that a selection has occurred during evolution. Many glycan structures are shared across Eukaryotes. The core structures identified for the main groups of glycans listed in Table 1 are found in all groups of Eukaryotes. This is true for *N*-glycans, *O*-glycans, C-mannose and glycosaminoglycans, in addition to glycolipids (see Kopitz 2017, this issue). Further elaboration of the core elements is achieved through pathways that are also shared across the Eukaryotes, but have been adapted to yield strain and phylum-specific glycans and provide a unique glycosylation pattern. The pathways necessary to achieve both core and peripheral glycans are also shared across the Eukaryotes and further underline the utilization of selective processes acting in evolution (Bertozzi and Rabuka 2009; Springer and Gagneux 2016).

It is also evident that differences exist between the phylogenetic groups comprising the Eukaryotes. The fungi are unable to synthesize proteins containing sialic acids, complex *N*-glycans, *O*-glycans glycosaminoglycans or single β-linked Glc*N*Ac. The green plants, Viridiplantae, do not synthesize sialic acids, phosphomannose units on high-mannose *N*-glycans, or β2-linked Glc*N*Ac on peripheral mannoses in *N*-glycans and generate plant-specific *N*-glycans with fucosyl and xylosyl units (Etzler and Mohnen 2009). *O*-Glycans are formed but show considerable differences to the Deuterostomes with no mucin-type proteins present and the main *O*-glycosylated products being hydroxyproline-rich glycoproteins (Wilson 2002). The nematodes also fail to synthesize sialic acids and do not form mucins. *N*-glycans are processed to yield paucimannose forms, while *O*-glycans are based on the core 1 disaccharide, but contain β-glucose, β-glucuronic acid and fucosylation patterns not seen in vertebrate glycosylation (Corfield and Berry 2015, supplemental material). Finally, the arthropoda synthesizes chitin as the major polymer found in the cuticular exoskeleton. Sialylation has been detected in *N*-glycans, but at low levels, in contrast to the vertebrates. *O*-glycans are also present, but appear to be limited to the core 1 disaccharide as no extended or branched glycans have been reported. In contrast, the Deuterostomes comprise the widest range of organisms sharing common glycan patterns (Corfield and Berry 2015, supplemental material).

## Glycosylation in cells

“Introduction” and “Glycosylation of proteins” sections serve to demonstrate that there are many examples where cellular glycosylation is employed to generate species-specific glycoproteins across the Eukaryotes. The mammals are the main source of data and form the basis for examples here. Mucosal surfaces throughout the mammalian body are adapted to provide function and communication with their specific environment. There is clearly a range of mucosae that can be identified, but only some of these have been examined in any detail. The examples given here serve to illustrate the basic properties and provide a basis for the reader to compare with the following other Eukaryotes and individual tissues where less information is available, starting with the glycocalyx (Habermann and Sinowatz 2009; Habermann et al. 2011; for details on the zona pellucida as an example for a glycocalyx, please see Manning et al. 2017).

As emphasized above, it is clear that glycosylation is present in the form of glycoconjugates throughout the cell. The cell surface has attracted most attention, as this is the interface where many crucial biological interactions occur. Glycosylation of proteins is the mechanism used by prokaryotes and Eukaryotes to form a base for recognition and other essential processes within the cell. These allow biological programming of proteins for selective functions. Examples include basic protein properties such as stability within defined biological environments (Lee et al. 2006; Saludes et al. 2010; Tran and Ten Hagen 2013), protein folding (Helenius et al. 1997; Petrescu et al. 2000; Aryal et al. 2010; Xu et al. 2013), intercellular trafficking (Lowe 1997; Huet et al. 2003), co-translational quality control (Helenius 2001; Xu and Ng 2015; Roth and Zuber 2017), protein maturation and half-life, also tested with synthetic glycans as signal to infer structure–activity relationships (Morell et al. 1971; Ashwell and Morell 1974; Jee et al. 2007; André et al. 1997, 2007; Unverzagt et al. 2002; Chen et al. 2012; Mi et al. 2014), mediation of cell interactions in the extracellular matrix (Bassaganas et al. 2014), host-microorganism recognition (Alemka et al. 2012; Hajishengallis et al. 2012) and cell–cell binding processes such as sperm–egg adhesion (Mengerink and Vacquier 2001; Velásquez et al. 2007; Pang et al. 2011).

All cells have an apical glycocalyx, which provides a dynamic barrier to allow communication with the external environment of each epithelial cell. This is a common feature across the Eukaryotes. This is a structural feature of the surface membranes and consists of an arrangement of glycoproteins and glycolipids as an array (see, e.g., Kesimer et al. 2013; Tecle and Gagneux 2015; Woods et al. 2015; Huang and Godula 2016). The glycans serve as recognition components for proteins that bind them, mediating many biological events, e.g. fertilization (Mourao 2007; Tecle and Gagneux 2015), embryogenesis (Baskin et al. 2010), tissue development and conservation (Hart and Copeland 2010; Wells et al. 2013), and including immune interaction at both the innate and adaptive levels (Paulsen 2008; Johansson and Hansson 2016) and disease progression. Individual glycan binding potencies are known to be weak; however, multivalent presentation, as a glycoside cluster in the glycocalyx, greatly reinforces the overall binding affinity and enhances discrimination. In addition, the levels of glycans present in the glycocalyx elicit responses in signalling pathways. Thus, specific densities of glycans in the glycocalyx can trigger cellular action through different signalling pathways.

**Fig. 4:**
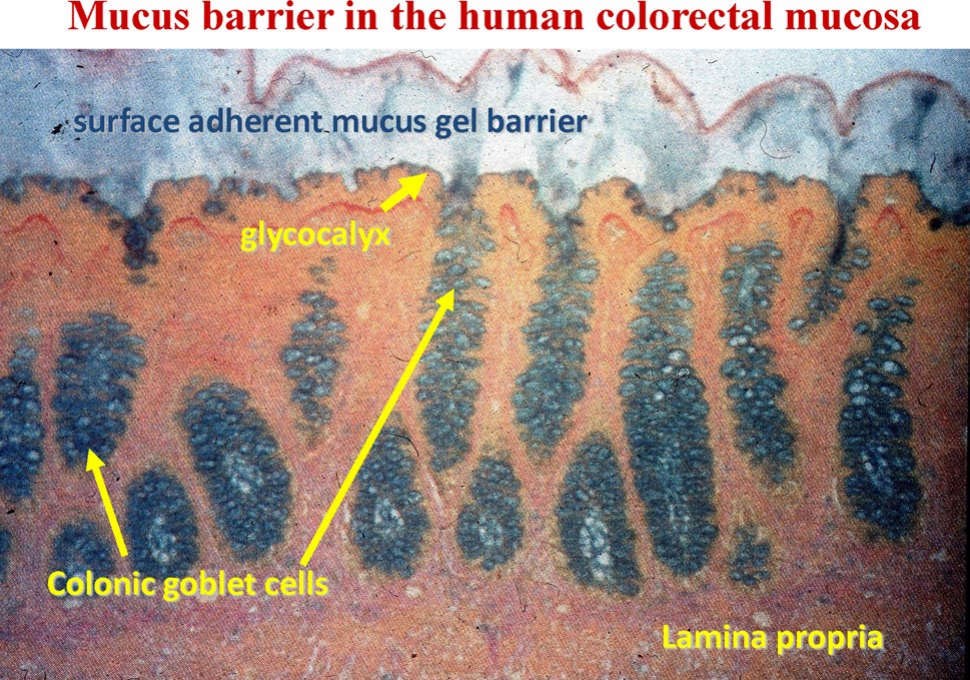
Intestinal mucus barrier. Mucosal sample stained histochemically with Alcian Blue and Van Gieson counterstain after stabilizing the mucus gel layer by cryostat and molten agar. The image shows the secreted gel layer, glycocalyx, goblet cells and lamina propria from human colon; from Pullan et al. (1994), with permission

Established histochemical and electron microscopic methods used to visualize the glycocalyx cause destruction of the mucus layer and disrupt the organization of the glycocalyx (Stonebraker et al. 2004; Ebong et al. 2011; Kesimer et al. 2013). Several improved techniques have been utilized to visualize the true thickness of the mucus layer, including the glycocalyx, in several mucosal systems (Pullan et al. 1994; Atuma et al. 2001; Strugala et al. 2003), see Fig. 4, and more recently under conditions where the dynamics of glycocalyx synthesis, mucus secretion and modulation of mucus thickness can be studied (Gustafsson et al. 2012). The glycocalyx is characterized by an abundance of membrane-associated mucins (MUC), such as MUC1, MUC4, MUC12, MUC16 and MUC20. These are expressed on a tissue-specific basis, although MUC1 appears to be common to most mucosal surfaces (Argüeso et al. 2003; Hattrup and Gendler 2008; Linden et al. 2008; Govindarajan and Gipson 2010; McGuckin et al. 2011; Corfield 2015).

It is important to emphasize that the mucosal surface epithelium throughout the mammalian body is comprised of a range of different cell types, each of which plays a role in general terms to provide a dynamic mucosal protective barrier (Gipson 2005, 2007; Johansson and Hansson 2013, 2016; Johansson et al. 2013; Birchenough et al. 2015). Mucus-producing cells have been identified in tissues where the secreted mucus layer is an essential feature of mucosal protection. The gastrointestinal epithelial cells that secrete mucus are the goblet cells, Tuft cells and Paneth cells. Other cells include the intestinal enterocytes and enteroendocrine cells, which are non-mucus-secreting cells. All of these cells are continuously renovated from stem cells located at the base of the crypt to maintain the proportions of these cells found under normal conditions. The intestinal enterocytes are the principal cells found in the intestinal mucosa and express many membrane-associated mucins on their glycocalyx. They are not concerned with significant secretion of mucus-type glycoproteins into the adherent mucus layer, but due to their abundance in the mucosal cell layer throughout the gastrointestinal tract their apical surface membrane domain makes a significant contribution to biological activity. Cell surface interactions are mediated through the glycan-rich zones of the mucins, which extend into the gastrointestinal lumen for a distance of up to 1 µm (Johansson and Hansson 2016). The mucins found have a typical pattern of expression throughout the whole intestine and may relate to general and specific biological roles. The precise pattern of glycans presented by these mucins throughout the intestinal tract is not well understood, but clearly links with function and remains an ongoing target for future research (Pelaseyed et al. 2014; Reunanen et al. 2015).

In the gastrointestinal tract, the interactions of the host with the resident microflora are regulated through immune system by presenting a range of antigens to allow maturation of lymphoid tissues. Peyer’s patches and the lamina propria are the sites where this occurs. Peyer’s patches have a characteristic dome shape and the M cells located in these regions phagocytose antigens to enable this process (Kraehenbuhl and Neutra 2000; Ermund et al. 2013a, b). The mucus layer at the surface of the Peyer’s patches is thought to be modulated, in order to allow easier sampling of bacteria. This may be due to the down-regulation of synthesis and secretion in those mucus-secreting cells bordering the Peyer’s patches, absence of mucus-secreting cells directly over the Peyer’s patches, or perhaps due to mucinolytic activities secreted by cells in the Peyer’s patches. However, this remains a controversial issue as dynamic mucus spreading and continuity along the surface lumen of the gastrointestinal tract is believed to occur. In addition, further experimentation is necessary to define the role of glycosylation in the recognition process, which mediates transfer of luminal material during sampling events.

Tuft cells, also known as brush cells, present as a fraction of small intestinal and respiratory tract epithelial cells and are responsible for sensing the microflora (Bezencon et al. 2008; Howitt et al. 2016). The location of Tuft cells as intestinal epithelial and respiratory tract, tracheal cells means that they will have direct contact with the parasites and microflora in the lumen of the gut and in respired air and therefore may contribute to homeostasis (Parfrey et al. 2014). These cells mediate type 2 immunity and are thought to recognize parasites. Some aspects of the glycobiology of Tuft cells have been examined (Gebhard and Gebert 1999), but there are no recent studies using modern methods. Furthermore, examination across the different phylogenetic groups comprising the Eukaryotes is only partly complete. These remain important aims to improve our understanding of cellular biology at all mucosal surfaces.

The gastrointestinal mucosal cells responsible for the synthesis and secretion of the mucus barrier are the goblet and Paneth cells. These two cell types form part of the single layer of epithelial cells found at mucosal surfaces. The Paneth cells are largely found in the small intestine and are closely linked with the synthesis and secretion of a range of inhibitors of bacterial growth, including the defensins (Bevins and Salzman 2011; Ouellette 2011; Clevers and Bevins 2013; Salzman and Bevins 2013). Paneth cells have been reported to secrete MUC2 (Johansson and Hansson 2016); however, there are no glycobiological data and the contribution to the mucus layer on the mucosal surface has not been assessed.

The goblet cells, named because of their shape, are typically identified due to the copious granules containing mucins, present in their apical region are abundant throughout the body. These cells have been identified in the salivary glands (Nieuw Amerongen et al. 1995; Tabak 2006; Rousseau et al. 2008; Kozak et al. 2016); the ocular surface in the conjunctiva (Gipson and Inatomi 1998; Gipson 2007); the oesophagus (Flejou 2005); the stomach (Reis et al. 1999); the duodenum (Buisine et al. 2000); the small intestine (Ermund et al. 2013a, b); the colorectum (Agawa and Jass 1990); the upper and lower airways (Rose and Voynow 2006; Thornton et al. 2008; Davies et al. 2012; Kreda et al. 2012); the male (D’Cruz et al. 1996) and female (Gipson 2001) reproductive tracts; the pancreas (Buisine et al. 2000) and the hepatobiliary system (Buisine et al. 2000).

The secreted mucus barrier is necessary to withstand the mechanical and physiological forces encountered in the intestine during peristalsis, to provide lubrication. It also provides innate and adaptive immunological protection (Johansson and Hansson 2016) and, furthermore, is designed to filter luminal material and nutrients and to interact with the microflora, including pathogens and parasites present in the gastrointestinal lumen (Hasnain et al. 2013; Johansson and Hansson 2016).

The mucus layer secreted by the goblet cells has a characteristic thickness and structure depending on the location of the mucosal surface. For example, in the gastrointestinal tract the thickness is greatest in the stomach and large intestine, typically around 700 µm, while the small intestine ranges between 150 and 300 µm (Atuma et al. 2001; McGuckin et al. 2011; Gustafsson et al. 2012). In the human colon a two-layer system is formed, the inner adherent layer composed of a network of MUC2 sheets, which is in contact with the mucosal cells and is resistant to penetration by the bacterial microflora (Johansson et al. 2008; Ambort et al. 2012). The outer layer is less organized and accommodates bacteria (Ambort et al. 2012).

**Fig. 5:**
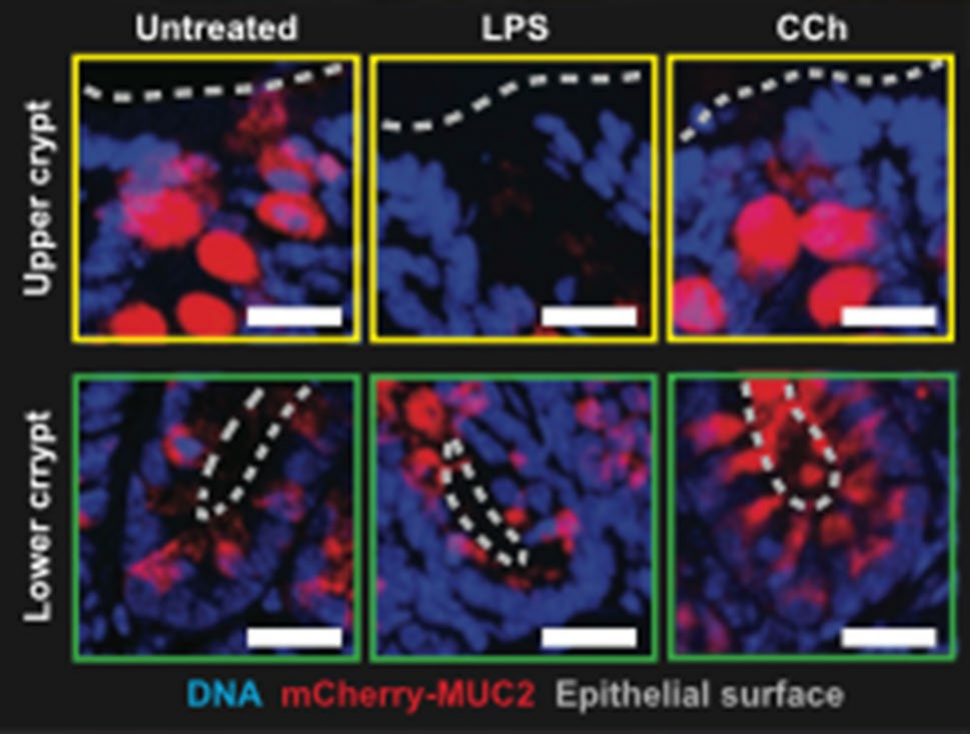
Sentinel goblet cells in the human colon. Goblet cells responsive to Toll-like receptor ligands (TLR ligands) are located in the upper crypt. Cryosections in colonic explants treated with TLR ligands and visualized by confocal microscopy. *Red* MUC2; *blue* DNA. Upper crypt (*yellow boxes*) or lower crypt (*green boxes*). A *dashed grey line* shows the epithelial surface. *Scale bars* 20 mm From Birchenough et al. Science 352:1535–1542 (2016). Reprinted with permission from the American Association for the Advancement of Science (AAAS)

Recent evidence has been presented that the goblet cells in the human colonic crypts are not equivalent (Birchenough et al. 2015, 2016). A sentinel goblet cell has been identified which is located at the entrance to the colonic crypt. The cell endocytoses TLR, which activates the Nlrp6 inflammasome, generates calcium-dependent MUC2 release from the sentinel cell itself and an intercellular gap junction signal. The signal leads to MUC2 secretion in neighbouring goblet cells in the upper crypt (Birchenough et al. 2015, 2016). This pattern of regulation ensures efficient protection against bacteria at the entrance to the crypt (Fig. 5).

Whether there are differences in the glycosylation of the MUC2 secreted at the surfaces of the crypts, from either the sentinel cell or those neighbouring goblet cells, has not yet been examined. The pattern of MUC2 glycosylation in goblet cells further down the crypt, which are not influenced by the sentinel cell, should also be considered. The picture that emerges is of a sophisticated defensive barrier system and not simply a MUC2 blanket.

Goblet cells produce a number of factors, which play a significant role in the regulation of mucus metabolism and in mucosal protection. These factors are linked to the synthesis of glycoproteins and have a role in glycobiological management (Rodríguez-Piñeiro et al. 2013; Pelaseyed et al. 2014; Johansson and Hansson 2016). The maturation of goblet cells is mediated by the action of the transcription factor SAM pointed domain-containing Ets transcription factor. Two goblet cell-specific ER proteins, anterior gradient protein 2 homologue (AGR2) and ER-to-nucleus signalling (ERN2 or IRE1β), are necessary for normal goblet cell MUC2 production (Johansson and Hansson 2016). The lectin-like protein ZG16 has been identified as an abundant goblet cell protein. It binds to the cell wall peptidoglycan of Gram-positive bacteria and leads to aggregation. These bacteria have reduced penetration of the mucus barrier at the colorectal surface, and ZG16 thus plays a role in keeping bacteria away from the mucosal surface (Bergström et al. 2016).

The trefoil factor family peptides are biosynthesized in the goblet cells and are closely linked to optimal organization of mucins and other glycoproteins in the secreted mucus barrier (Wright 2001; Hoffmann 2004; Albert et al. 2010). Resistin-like molecule is a cysteine-rich protein specifically produced by intestinal goblet cells and is thought to function in the mucosal barrier through regulation of inflammation (He et al. 2003; Artis et al. 2004; Wang et al. 2005). It has been shown to lead to colitis by depleting protective bacterial strains in the gut microflora (Morampudi et al. 2016).

The oral cavity and salivary glands are the initial link with the oesophagus and gastrointestinal system. The salivary glands have been well studied, and information regarding the range of mucins and salivary proteins with their glycobiology is extensive (Veerman et al. 2003; Tabak 2006; Nieuw Amerongen et al. 2007; Tian and Hagen 2007; Rousseau et al. 2008; Kozak et al. 2016).

In the respiratory tract, the pseudostratified, ciliated and columnar tracheal and bronchiolar epithelial lining includes basal cells, secretory cells and ciliated cells. Ciliated cells are the most abundant, while goblet cells show a regional distribution being more numerous in the trachea than the bronchioles. The cells that secrete mucus are the goblet cells and mucus-small granule cells. In addition, the submucosal glands contribute a major part of secreted tracheobronchial mucus. They are abundant in the larger bronchi and have typical morphology with mucous and serous acini, a collecting duct and tubules and a ciliated duct. The major glycoproteins synthesized in the respiratory tract are the mucins (Andrianifahanana et al. 2006). In man the main secreted mucins are MUC5AC, found exclusively in the epithelial goblet cells and MUC5B synthesized in the submucosal glands and associated ducts (Buisine et al. 1999; Kirkham et al. 2002; Sheehan et al. 2004; Voynow et al. 2006; Rousseau et al. 2007; Thornton et al. 2008). Low levels of MUC2 are produced in some goblet cells and the basal cells, while MUC7 is produced in the serous cells (Buisine et al. 1999; Copin et al. 2000; Vinall et al. 2000). The membrane-associated mucins MUC1 and MUC4 are detected in the tracheal epithelium (Hattrup and Gendler 2008), and expression of MUC3 (Apostolopoulos et al. 1995), MUC13 (Williams et al. 2001), MUC19 (Chen et al. 2004) and MUC20 (Higuchi et al. 2004) has been found. The molecular and physiological significance of this array of mucins remains to be clarified and the glycobiological data are limited, although characteristic glycosylation patterns for the respiratory tract are expected (Thornton et al. 1997, 1999, 2000; Holmén et al. 2004; Kesimer et al. 2013).

The human reproductive tract in both men and women has been a focus of attention, especially with regard to fertilization. However, it also provides a specific epithelial environment enabling the fertilization process and simultaneously supporting mucosal protection. As major glycoprotein components at animal mucosal surfaces, the mucins and sialoglycoproteins are prominent in male and female reproductive tracts (Audié et al. 1993; DeSouza et al. 1998; Lewis and Lewis 2012). There is ample evidence that the glycosylation of these molecules is an important factor for these molecules and plays a functional role in a number of different ways. This underlines, again, the flexibility of glycosylation as a dynamic and expansive mechanism adapted to specific physiological requirements. The physicochemical properties of the mucins are generated through the high proportion of glycans in these molecules (Lewis and Lewis 2012). A protective role for the mucins in the oviduct has been demonstrated with regard to both the migration of spermatozoa and the movement of fertilized ova to the uterus (Jansen 1995). Furthermore, manipulation of glycan chains through the action of mucinases and glycosidases such as sialidases plays both general and specific roles in man (Wiggins et al. 2001) and other Eukaryotes, including monotremes (Oftedal et al. 2014) and fish (Hunt et al. 2005).

**Fig. 6:**
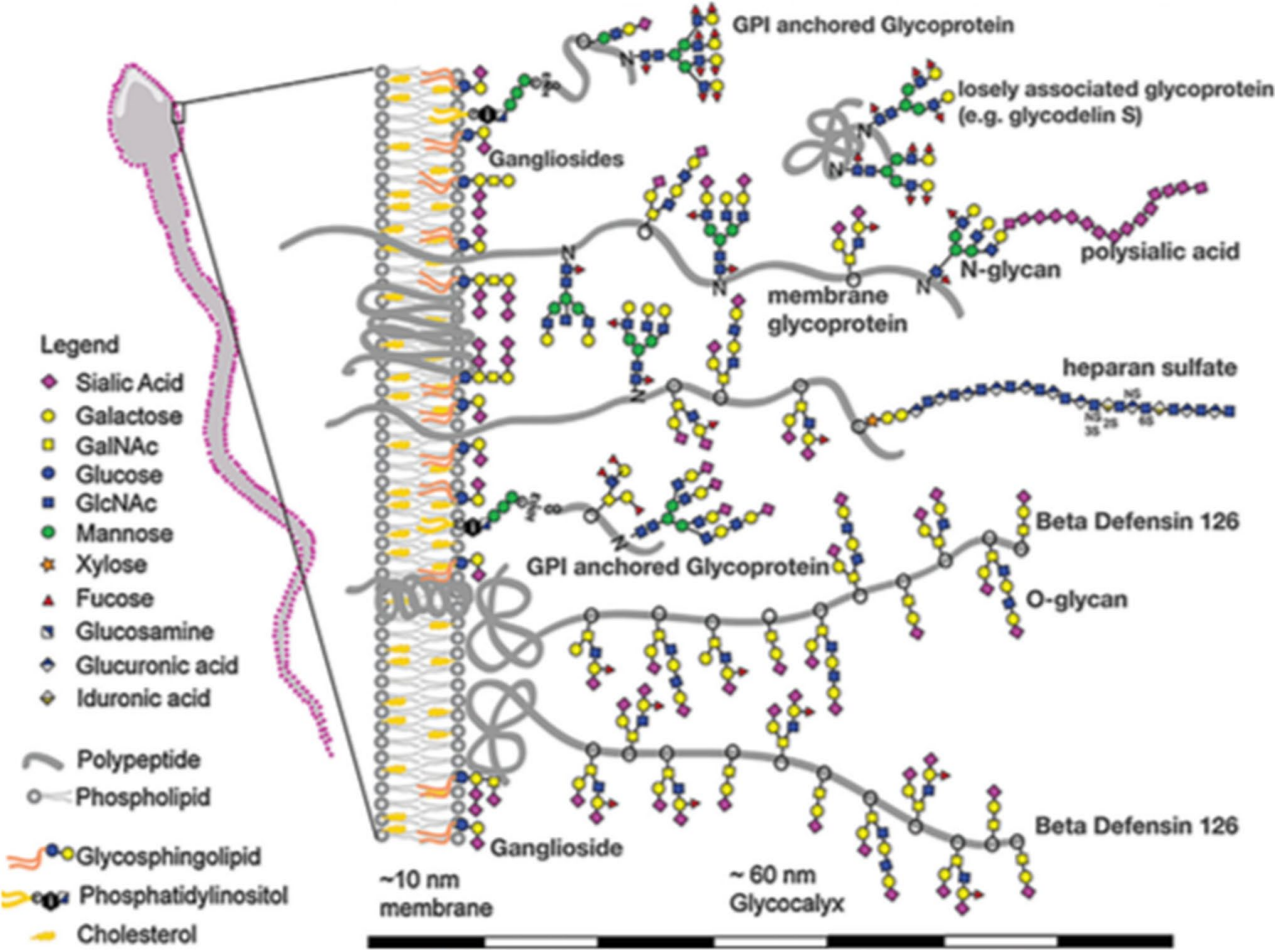
Major glycan and glycoconjugate classes of human sperm glycocalyx. Monosaccharides are coded by *coloured symbols* shown in the figure. Proteins and lipids are *grey*, except cholesterol, and the lipids of glycosphingolipids. Mammals synthesize most glycans with a dozen different monosaccharide-building blocks; some of these monosaccharides can be further modified by sulphation and/or acetylation; from Tecle and Gagneux (2015), with permission

The main partners in Eukaryote fertilization, the sperm and the egg have been studied extensively and functions for glycosylation clearly identified (Tecle and Gagneux 2015). Spermatozoa have been investigated across the spectrum of Eukaryotes and possess an abundance of glycoconjugates on their surface membranes, the glycocalyx, which extends for 60 nm from the membrane (Fig. 6). Among the glycoconjugates in the sperm glycocalyx are typical membrane glycoproteins with membrane anchor peptide domains together with glycoproteins anchored by a glycophosphatidylinositol unit (Franke et al. 2001; Mengerink and Vacquier 2001; Koistinen et al. 2003; Klisch et al. 2011; Tecle and Gagneux 2015). The glycobiology of these glycoconjugates has been defined (McCauley et al. 2002; Diekman 2003; Parry et al. 2007; Velásquez et al. 2007; Tecle and Gagneux 2015), and sialic acids play important roles (Yudin et al. 2008; Tollner et al. 2012; Silva et al. 2014). Important events in sperm–egg binding are mediated through the glycans on these molecules. Sialyl-Le^x^ has been identified as a partner in sperm–egg binding (Pang et al. 2011).

Maturation of the sperm glycocalyx is necessary for penetration of the mucus barrier in the cervix and also provides protection against uterine immune defences. Both sialylation (Mengerink and Vacquier 2001; Miyata et al. 2004, 2006; Velásquez et al. 2007; Ma et al. 2012; Simon et al. 2013; Hänsch et al. 2014) and fucosylation (Mengerink and Vacquier 2001; Pang et al. 2007; Tecle and Gagneux 2015) play roles in the development, maturation and functional aspects of spermatozoa. The range of sialic acids has been shown to act as self-associated molecular patterns and are binding partners for proteins synchronizing the immune response such as the siglecs (Gabius 1997; Crocker 2005; Varki 2011). The migration of spermatozoa to the oviduct involves glycocalyx interactions with the follicular fluid and epithelial barrier of the uterus, leading to the formation of the oviductal sperm reservoir (Tecle and Gagneux 2015) and ultimately binding to the zona pellucida of the oocyte. Sperm capacitation occurs before fertilization and is necessary to allow the normal fertilization process to occur. This entails a substantial reorganization of the glycocalyx. Membrane-anchored glycoproteins are discarded, and specific desialylation occurs (Ma et al. 2012; Tollner et al. 2012; Tecle and Gagneux 2015).

The female reproductive tract mucosal cells have a typical glycocalyx and secrete a variety of glycoproteins in a hormonally mediated fashion. This leads to variation in thickness and porosity of the surface mucus gel, which correlate with sperm penetration and fertilization of the ova. Mucins and glycodelins are important and have glycoforms that vary throughout the menstrual cycle and accommodate the processes occurring during fertilization. Mucins identified include the secreted forms MUC5AC, MUC5B and MUC6, together with membrane-associated MUC1 and MUC16 (Gipson et al. 2001; Argüeso et al. 2002; Andrianifahanana et al. 2006; Andersch-Bjorkman et al. 2007; Gipson et al. 2008; Pluta et al. 2012; Corfield 2015). Over 50 *O*-glycans were detected including neural and acidic with both sialylated and sulphated forms. Ovulation was characterized by decreased sialylation and an increase in core 2 structures, while Neu5Acα2,6Gal*N*Ac- and Neu5Acα2,3Gal-glycans were common in the non-ovulatory phases (Yurewicz et al. 1987; Andersch-Bjorkman et al. 2007).

A report on the action of hormones and bacterial flora on the female genital tract glycome during the menstrual cycle has recently appeared (Moncla et al. 2016) and identified MUC1, MUC4, MUC5AC and MUC7 with distinct glycosylation patterns identified using lectins (Moncla et al. 2016). This study demonstrates organized expression of both mucins and glycosylation during the menstrual cycle. The glycodelins are glycoproteins also found in the female reproductive tract. They are small, 28- to 30-kDa glycoproteins of the lipocalin family and occur as three isoforms, each with two *N*-glycan chains (Seppala et al. 2002; Jayachandran et al. 2006; Yeung et al. 2006). Complex-type glycans were detected: Galβ1,4Glc*N*Ac, Gal*N*Acβ1,4Glc*N*Ac, NeuAcα2,6Galβ1,4Glc*N*Ac, NeuAcα2,6Galβ1,4Glc*N*Ac, Galβ1,4(Fucα1,3)Glc*N*Ac and Gal*N*Acβ1,4(Fucα1,3)Glc*N*Ac (Dell et al. 1995). There is evidence that glycans with sialyl-Lac*N*Ac or Lacdi*N*Ac antennae play a role in immunosuppression, through CD22 and selectins (Dell et al. 1995). Sperm–zona pellucida binding is blocked by glycodelin and further emphasizes the importance of glycobiology in immune and gamete recognition processes (Dell et al. 1995).

In addition to the secretions associated with the mucosal surface, the cervical canal contains a mucus plug during pregnancy. Although this is a well-known mucus feature, it has received little interest compared to the other mucosal surfaces in the body (Becher et al. 2009). Most interest has focused on the rheological (Becher et al. 2009; Bastholm et al. 2016), microbial (Hansen et al. 2014), immunological (Hein et al. 2005; Lee et al. 2011) and protein degradative (Becher et al. 2010) properties. Protein profiling studies showed MUC1, MUC2, MUC5AC and MUC5B (Habte et al. 2008), but did not identify glycodelins (Habte et al. 2008; Lee et al. 2011). No glycan analysis of the mucus plug glycoproteins has been reported.

The jelly coat or extracellular matrix surrounding Deuterosome eggs is species-specific and linked to the process of sperm–egg fusion to achieve fertilization. While the echinoderms have a jelly layer and vitelline coat, mammals have a more complex arrangement with an external cumulus matrix overlying a zona pellucida (Mengerink and Vacquier 2001; Habermann and Sinowatz 2009; Habermann et al. 2011).

**Fig. 7:**
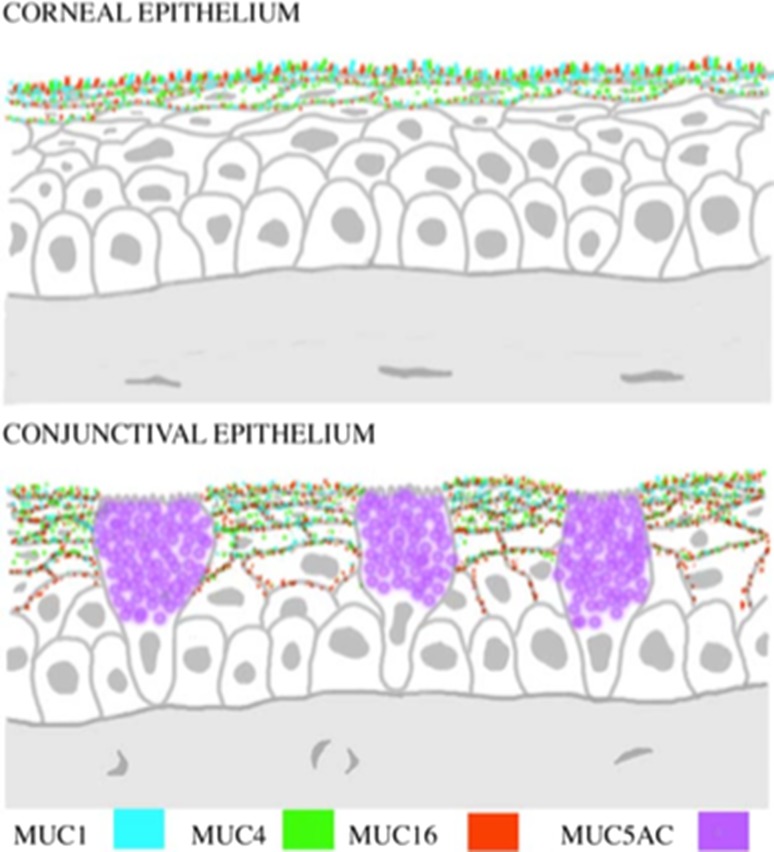
Mucin expression in the human cornea and conjunctiva. Schematic of the location of mucins in the corneal and conjunctival epithelium. The membrane-associated mucins MUC1, MUC4 and MUC16 at the apical cell membrane glycocalyx, and the secreted mucin MUC5AC in goblet cell vesicles; from Gipson (2004), with permission

The ocular surface and tear film is a specially adapted mucosal surface which has properties and structures not seen at other mucosal locations. The conjunctiva is the mucosal surface where products composing the tear film are synthesized and secreted and together with the underlying stroma provides ocular defence and protection (Fig. 7). The conjunctival epithelium is composed of squamous and goblet cells which both secrete electrolytes. The mucins constitute the major molecules contributing to the structure and properties of the tear film (Berry et al. 1996; Pflugfelder et al. 2000; Gipson 2004, 2007; Paulsen 2008; Argüeso and Gipson 2012; Hodges and Dartt 2013). They address several biological requirements of the ocular surface. Physicochemical properties enable lubrication during blinking and spreading across the corneal and conjunctival surfaces, allow removal of debris accumulated on the eye surface and retain hydration to avoid dessication. These physicochemical properties are also designed to allow light through the barrier to give optimal vision. Mucins generate a stable gel layer where anti-microbial molecules, including lysozyme, transferrin, secretory IgA and other immunoglobulins, defensins and trefoil factor family peptides, can be maintained to achieve protection against infection (Gipson and Argüeso 2003; Argüeso et al. 2009; Govindarajan and Gipson 2010).

The tear film is composed of three layers, an apical surface lipid layer, secreted by the Meibomian glands, an aqueous layer lying above the mucus layer, which has direct contact with the glycocalyx at the apical surface of cornea. The tear film is a dynamic entity, and each layer is constantly renewed.

The ocular mucins show a selective expression pattern, again emphasizing the adaptation of mucosal surfaces to environmental needs (Young and Clement 2000). MUC5AC is the major secreted mucin present, while MUC2, MUC5B, MUC7 and MUC19 have been detected at lower levels (Berry et al. 1996; Gipson 2004; Mantelli and Argüeso 2008). The glycocalyx of the stratified epithelium is rich in membrane-associated mucins, MUC1, MUC4 and MUC16 are most abundant (Argüeso et al. 2003; Paulsen and Berry 2006; Govindarajan and Gipson 2010; Hodges and Dartt 2013), and MUC15 and MUC20 have also been detected (Mantelli and Argüeso 2008). The lacrimal glands also contribute to the composition of tear film and MUC1, MUC2, MUC4, MUC5AC, MUC5B, MUC6 and MUC7 have been detected in the glands and the secreted mucins MUC5AC, MUC5B, MUC6 and MUC7 probably reach the tear film through the tear duct passage (Paulsen and Berry 2006).

The glycobiology of the ocular surface and tear film has been probed in several studies, where the significance of sialic acids is a common characteristic (Pflugfelder et al. 2000; Corfield et al. 2005; Argüeso and Sumiyoshi 2006; Argüeso 2008; Royle et al. 2008; Argüeso et al. 2009; Baos et al. 2012). Examination of the *O*-linked glycans released from the mucins in human, rabbit and canine ocular surface secretions revealed 12 different glycans, 6 of which were sialylated (Royle et al. 2008). Further chemical, lectin and antibody studies demonstrated that the 9-*O*-acetylated form of sialic acid was a characteristic feature of the ocular system (Corfield et al. 2005; Argüeso and Sumiyoshi 2006). Thus, a tissue-specific glycosylation programme operates at the ocular surface and emphasizes the versatility of the sugar code as a means to achieve biological specificity. Imaging of ocular mucins using atomic force microscopy has enabled mapping of the height of glycans on MUC5AC through binding of an anti-glycan antibody (Round et al. 2007). These data support proposals regarding the arrangement of mucin molecules in aqueous solution under physiological conditions.

The urinary tract is another example of a tissue where the mucosal surfaces have been adapted to allow specific function. Filtration of urine requires barrier properties not found at other mucosal sites. The renal mucins and kidney-specific glycoproteins are well known (Serafini-Cessi et al. 2005; Aubert et al. 2009; Tringali et al. 2012; Weinhold et al. 2012). Especially with respect to sialic acid metabolism in glomerulus podocytes (Wagner and Roth 1985; Charest and Roth 1988).

The nervous system has attracted considerable attention and exhibits a characteristic pattern of molecular morphology having an array of tissue-specific molecules with characteristic glycosylation, as further described by Ledeen and Wu (2009) and by Higuero et al. (2017, this issue). A short overview is given here. Neural cell adhesion molecule (Zhou and Zhou 1996) is a member of the immunoglobulin superfamily of adhesion molecules and carries polysialic acid chains of varying size. These polysialic acid chains are α2–8 linked and have biological role in nervous tissue and especially the brain (Hildebrandt et al. 2007; Rutishauser 2008; Bonfanti and Theodosis 2009; Mühlenhoff et al. 2009; Zuber and Roth 2009; Schnaar et al. 2014). Neural stem cells express CD15 (Yu and Yanagisawa) coding Galβ1,4(Fucα1,3)Glc*N*Acβ1- (Yu and Yanagisawa 2006), while *O*-mannosylation is also a significant feature of α-dystroglycan in the nervous system, where it mediates cell-extracellular matrix contact (Hennet 2009; Panin and Wells 2014; Praissman and Wells 2014; Yaji et al. 2015).

The innate and adaptive immune systems have been extensively scrutinized regarding their glycobiology (Bäckhed et al. 2001; Royle et al. 2003; Bevins 2004; Rudd et al. 2004; Brockhausen 2006; Crocker et al. 2007; Marth and Grewal 2008; Hooper et al. 2012; Kolarich et al. 2012; Rabinovich et al. 2012; Bull et al. 2014; Gerbe and Jay 2016; Johansson and Hansson 2016).

## Glycosylation and disease

There are many examples of aberrant glycosylation playing a role in disease processes. This part uses a few examples to highlight the relationship between incorrect glycosylation, biological recognition and the resulting changes that lead to abnormal function and pathology. The techniques employed to detect changes in protein glycosylation are those outlined in “Glycosylation of proteins” section.

The development of cancer in the human gastrointestinal tract has been closely studied as it falls into the category of a Western disease, linked with lifestyle and diet. Much work has focused on the mucosal changes associated with the development of the tumours, and inflammatory bowel disease (IBD) has proved to be instructive due to the number of patients who go on to develop cancer. From early days there have been indications that the changes in glycobiology are related to the process of malignant transformation. The routine histological screening of the gastrointestinal tract for early changes in disease remains an essential part of clinical assessment. Early detection is associated with positive prognosis, and regular screening during disease provides assessment of disease progression and gives indications for therapy including surgical intervention. The focus of this section is the lower bowel, the colorectum and the patterns associated with progression to cancer. Important chemical and biochemical information has been gathered regarding the pattern of glycosylation in mucins at different regions of the human colorectum (Robbe et al. 2003, 2004, 2005; Larsson et al. 2009; Holmén Larsson et al. 2013) and similarities that exist compared to the foetal mucins (Robbe-Masselot et al. 2009). However, differences between MUC2 from human and murine colorectum have been shown and underline the need to take account of species-specific glycosylation when studying disease mechanisms (Thomsson et al. 2012). These data are important as it provides a chemical basis to consider the lectins and antibodies, which are valuable in screening tissue sections from patients.

The inflammatory bowel diseases, ulcerative colitis (UC) and Crohn’s disease (CD), show characteristic morphological changes at the sites of disease, and colorectal tumours are also found at these sites. Endoscopic screening is used to locate regional mucosal disease along the colorectum, and both UC and CD have characteristic patterns of disease mucosa flanked by normal mucosa. Colonic biopsies taken at sites of disease together with resected tumours and polyps have been used for histological assessment.

Because the mucins expressed throughout the colorectum present the main glycan repertoire, tissue samples have been examined for both MUC gene expression and glycosylation patterns (Bartman et al. 1999; Kyo et al. 2001; Sylvester et al. 2001; Shaoul et al. 2004; Longman et al. 2006; Png et al. 2010; Corfield et al. 2011; Croix et al. 2011; Larsson et al. 2011; Sheng et al. 2012; Leone et al. 2013; Theodoratou et al. 2014).

**Fig. 8:**
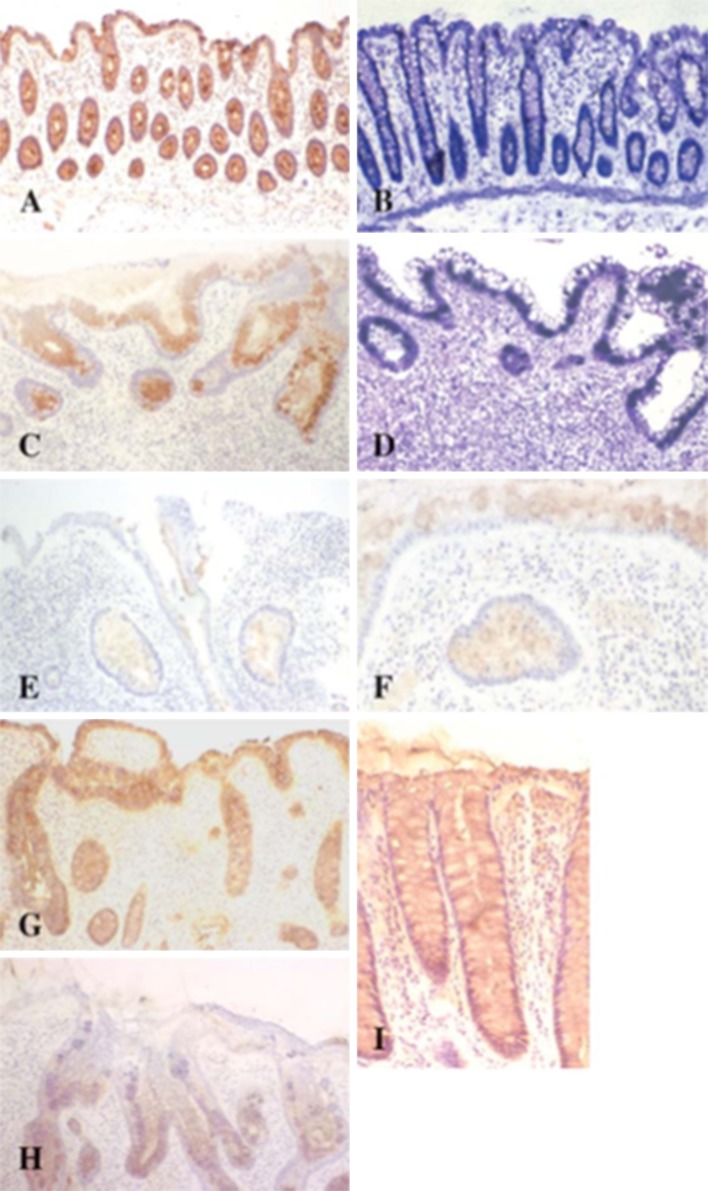
Loss of MUC2 and sulpho-Lewis^a^ in ulcerative colitis. Detection of MUC2 glycoprotein by immunostaining with the Lum2–3 antibody for normal (**a**) and clinically severe UC (**c**). The same specimens were also tested for MUC2 mRNA for normal (**b**) and UC (**d**). Immunostaining for MUC2 in mucosa adjacent to an ulcer (**e**) showed reduced staining compared with normal intact mucosa (**f**). Detection of sulpho-Le^a^ with the F2 antibody showed localization throughout the mucosa, including the mucus gel layer (**g**). Sulpho-Le^a^ staining was preserved in mild colitis (**i**), but was depleted at the luminal surface and upper crypts in severe colitis (**h**). In situ hybridization samples were counterstained with toluidine blue and immunohistological sections with haematoxylin; from Longman et al. (2006), with permission

The screening of mucins in the colorectum has identified MUC2 as the major product (Tytgat et al. 1994; van Klinken et al. 1997). Confirmation of the relevance of this gene to colorectal disease has come from Muc2^−/−^ mice where the gene is deleted and increased susceptibility to spontaneous tumour formation occurs (Velcich et al. 2002). Other mucin changes in colorectal cancer have been reported for *de novo* expression of MUC5AC (Buisine et al. 1996, 2001; Myerscough et al. 2001; Kocer et al. 2002; Warson et al. 2002) and MUC 20 overexpression (Xiao et al. 2013). A number of studies have addressed the glycosylation of mucins in both IBD and colorectal cancer and the progression through different stages from adenoma to carcinoma (Fig. 8). The importance of the attachment of the *O*-glycans to mucin proteins was demonstrated with studies on the core 1 disaccharide Galβ1,3Gal*N*Ac-, also called TF antigen (Campbell et al. 2001; Bergström et al. 2016), and the core 3 Glc*N*Acβ1,3Gal*N*Ac structures (Iwai et al. 2005). Loss of genes coding for the formation of these cores led to development of colitis and cancer. Malignant change leads to a reduction in the size of the glycans on mucins and an enrichment of the truncated glycans, Tn (Gal*N*Ac-α-Ser/Thr), sialyl-Tn (Neu5Acα2,6Gal*N*Ac-α-Ser/Thr) (Itzkowitz et al. 1990; King et al. 1994; Jass et al. 1995; Brockhausen et al. 1998; Marcos et al. 2011) and the TF antigen Galβ1,3Gal*N*Ac-αSer/Thr (Baldus et al. 2000; Campbell et al. 2001). Interestingly, these epitopes are targets of diverse tissue lectins such as C-type lectins as the macrophage receptor, with different impact on the fate of the tumour cells ranging from defence to growth stimulation (Marcelo et al. 2014; Rodriguez et al. 2015; Beatson et al. 2016). Changes in mucin sialylation are also evident with an increase of sialyl-Le^x^ (Hanski et al. 1995; Grabowski et al. 2000; Robbe-Masselot et al. 2009) and the loss of the Sd^a^ antigen, Gal*N*Acβ1,4(Neu5Acα2,3)Galβ1,3/4Glc*N*Acβ1,3Gal*N*Ac- (Malagolini et al. 2007). Variation of sialytransferases in colorectal cancer accounts for these alterations (Dall’Olio et al. 1989, 2014). High levels of *O*-acetylated sialic acids are normally present in colonic mucins and are depleted in IBD and colorectal cancer (Corfield et al. 1992a, 1999; Mann et al. 1997; Shen et al. 2004). Changes in the *O*-glycan pattern for MUC2 have been reported in UC (Larsson et al. 2011).

Glycosulphation is a major feature of colonic mucins and is reduced in IBD and cancer. This has been shown using histochemical staining by the high iron diamine method and metabolic labelling with ^35^S-sulphate (Corfield et al. 1992b, 1996; Campbell et al. 2001). Immunohistochemistry showed a reduction in sulpho-Le^a^, SO_4_-3Galβ1,3(Fucα1,4)Glc*N*Acβ1,3Gal-staining (Longman et al. 2006).

Strong evidence supports a role for the microflora in the pathology of IBD and colorectal cancer (Knight et al. 2008; Png et al. 2010; Arthur and Jobin 2011; Candela et al. 2011; Fava and Danese 2011; Gentschew and Ferguson 2012; Natividad et al. 2012; Dalal and Chang 2014; Probert et al. 2014), and therapeutic faecal transplant is currently being used (Shanahan and Collins 2010; Damman et al. 2012). These aspects also link with the influence of the diet on disease (Albenberg et al. 2012; Neuman and Nanau 2012). Thus, interactions of the microflora in the gut with the host mucosa impact on the metabolic pathways regulating mucins and their glycosylation. Ethnicity has been found to be a factor in the nature of IBD and colorectal cancer. The lower rate of IBD in Indian populations compared with Caucasians could be confirmed using histological and metabolic labelling methods (Probert et al. 1993, 1995). This remains an underdeveloped aspect of gastrointestinal disease worldwide.

**Fig. 9:**
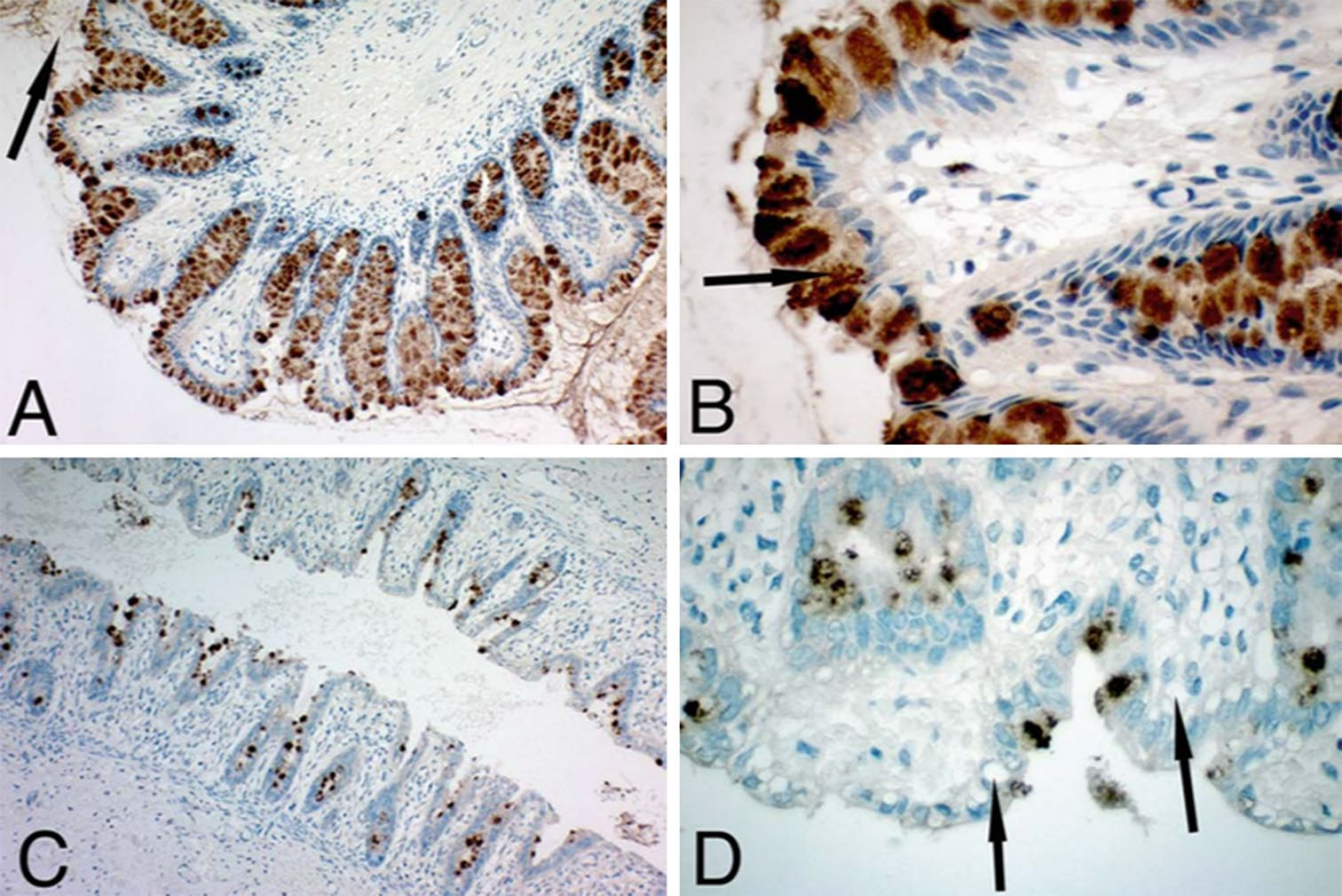
Depletion of goblet cells in necrotizing enterocolitis. The trefoil factor family peptide TFF3 is shown with immunostaining (**a**) in normal neonatal colon (×10 magnification), with staining of all goblet cells and the lumen (*arrow*). Normal neonatal colon at ×40 magnification shows a granular pattern of TFF3 in goblet cells (**b**). Reduction of goblet cells in a patient with NEC is shown at ×10 (**c**) and ×40 (**d**) magnification and reveals empty goblet cells (*arrows* in **d**), in particular at the surface epithelium; from Vieten et al. (2005), with permission

Necrotizing enterocolitis is the most common gastrointestinal condition known for premature, low-birth-weight neonates. It has a multifactorial aetiology, and its pathogenesis remains poorly understood. A combination of risk factors results in the attachment of bacteria to the immature and damaged mucosal barrier mucus. Maintenance of the cellular integrity of the mucosal barrier is crucial and correlates with a number of factors, including the trefoil factor family peptides, found in the goblet cells and which are responsible for regulating epithelial homeostasis through restitution and regeneration. Together with the mucins they provide effective mucosal barrier protection at this early stage of life. The glycobiology of this condition has not been examined, but demonstration that depletion of goblet cell TFFs occurs (Vieten et al. 2005), in this condition implies a role for in this condition (Fig. 9). In view of the better understanding of goblet cells and the existence of different types with specific roles, noted above under “goblet and Paneth cells and the secreted mucus layer”, a closer examination of this phenomenon in NEC may lead to improved understanding of pathogenesis and protective measures.

Bacterial vaginosis is a common polymicrobial condition found in women. In women of childbearing age the vagina is host to an abundance of bacterial strains dominated by *Lactobacillus* species (Onderdonk et al. 2016). The degradation carbohydrate by these bacteria produces lactic acid, which maintains a vaginal surface pH of around 4.5, which is optimal for their growth. In BV an increase in mucosal pH to 6.0–7.0 lead to a change in the microflora with a loss of *Lactobacillus* spp. and the appearance of other facultative and anaerobic strains including anaerobic cocci, *Gardnerella vaginalis*, *Mobiluncus* spp., *Bacteroides* spp., *Prevotella* spp., *Peptostreptococcus* spp. and *Mycoplasma hominis*. Clinically, this change leads to poor health outcomes including pelvic inflammatory disease, late miscarriage, spontaneous preterm birth and chorioamnionitis. Probiotic therapy has been used in disease management (Falagas et al. 2007). The cervicovaginal fluid (Wang et al. 2015) is part of the mucosal lining of the vagina and is analogous to the mucus barrier in other mucosae (Moncla et al. 2016). Cervical mucins and a range of other defensive proteins are present in the secretion and donate the major glycan composition present in this environment (Gipson 2001; Pluta et al. 2012). The secretory mucin genes MUC2, MUC5AC, MUC5B, MUC6 are found with major expression of MUC5AC and MUC5B (Gipson et al. 1999, 2001), while the membrane-associated mucins, MUC1, MUC4 and MUC16 are found at high levels (Gipson et al. 1999, 2001). Glycobiology plays a significant role in the pathology of BV. The action of mucinases and glycosidases, especially sialidases (Wiggins et al. 2000), has been demonstrated to be associated with the development of an abnormal mucosal barrier in the vagina (Wiggins et al. 2001; Moncla et al. 2015). In addition, the association of glycosulphatases in BV microflora has been found and implicates mucin sulphation as a feature of the normal and pathological state (Roberton et al. 2005). Review of the female genital tract glycome has confirmed this relationship and yielded a focus for future development and therapeutic intervention (Moncla et al. 2016).

The ocular surface and the tear film have been widely examined in mammals as detailed above under “The Ocular Surface”. Among pathological conditions that affect the normal function is dry eye disease or keratoconjunctivitis sicca. In this disease, a number of morphological abnormalities of the lacrimal apparatus arise which cause instability and dessication of the preocular tear film and changes in the ocular surface mucus. A reduced number of conjunctival goblet cells are detected histologically (Vieten et al. 2005), and the mucus becomes more viscous and less easy to spread evenly over the corneal surface (Corfield et al. 2005).

**Fig. 10:**
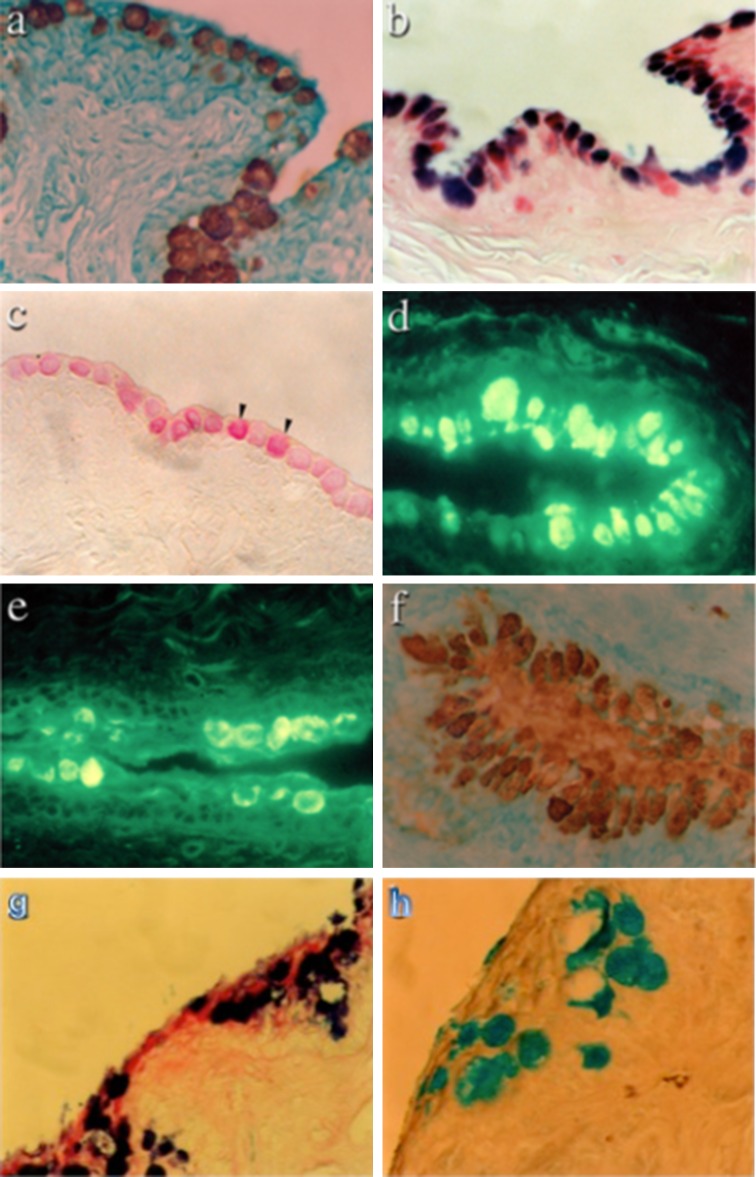
Histology of normal and KCS canine conjunctival tissues. Tissue sections from normal canine conjunctiva were stained with the anti-MUC5AC antibody 21M1 (**a**); Alcian Blue/PAS (**b**); mild-PAS (**c**); SNA for α(2–6)-linked sialic acids (**d**); WGA (**e**) and antibody TKH2 against sialyl-Tn (sialyl-α(2–6)GalNAc) (**f**). Magnification is ×40 in all cases. Histology of KCS canine conjunctival tissues. Tissue sections from animals with KCS stained with periodic acid-Schiff/Alcian Blue (**g**) and high iron diamine/Alcian Blue (**h**). Magnification is ×40 in both cases. Taken from Corfield et al. (2005) with permission

Studies show that glycobiology plays a significant role in this disease (Argüeso 2008). Sialylated *O*-glycans were detected in human and canine mucus (Royle et al. 2008; Guzman-Aranguez et al. 2009) and a reduction in the expression of sialyl-Le^a^ reported in dry eye tears (Garcher et al. 1998). Although increased sialylation was found in canine KCS (Carrington et al. 1998), the fraction of these sialic acids present as 9-*O*-acetylated sialic acid, largely in MUC 5AC mucins, was reduced or eliminated in KCS (Gipson et al. 2004; Corfield et al. 2005; Argüeso and Sumiyoshi 2006) (Fig. 10). These data emphasize the importance of glycobiology in understanding biological function.

The presence of amyloid deposits in the brain is a hallmark of Alzheimer’s disease (AD) (Shoemark and Allen 2015) and increases with age in humans and is associated with progressive decline of cognitive function and dementia. The pathogenesis of AD is still poorly understood, but the identification of the accumulation of protein aggregates derived from amyloid precursor protein has been identified. The normal proteolytic processing of APP is implicated in AD, and the resulting aggregates form a focus for the deposition of amyloid to generate senile plaques in the brain. Correlations of AD with the patterns of microbial colonization in the body and changes associated with increased age have been presented (Shoemark and Allen 2015), and these may link with glycoprotein metabolism through interactions at mucosal surfaces throughout the body, especially in the gastrointestinal tract, as discussed above. In support of this suggestion, recent data in a mouse KO model for neuraminidase 1 (Neu1) *Neu1* ^−/−^ have demonstrated a role for this gene. Under normal conditions, this gene codes for an enzyme that desialylates sialoglyconjugates. This enzyme is known to regulate lysosomal exocytosis through its action on the lysosome-associated membrane protein 1 (Yogalingam et al. 2008). One function of this protein is the mediation of lysosome docking at the plasma membrane. These lysosomes then release their contents into the extracellular environment. This function is common to the majority of cells. In the absence of NEU1, the lysosomal-associated membrane protein 1 (LAMP-1) remains sialylated and leads to an increased number of lysosomes at the plasma membrane. As a result abnormal remodelling of the extracellular matrix and plasma membrane glycocalyx occurs (Annunziata et al. 2013). The work identifies APP as a substrate for NEU1 and rescue of the condition in *Neu1* ^−/−^ mice is achieved by intracranial injection of NEU1, resulting in fewer amyloid plaques (Annunziata et al. 2013). These results open the door for further examination of glycosylation in AD and potential therapeutic approaches.

## Conclusions and perspectives

This review provides an overview of the range of glycan structures present in and utilized by the Eukaryotes. Future studies will be in the application of structural technology and bioinformatics to clarify the glycosylation of glycoproteins structurally and conformationally. This is especially important for those situations, where glycoproteins have an already clearly defined biological function and also where changes in glycosylation occur in relation to normal development, tissue function and disease. Attractive perspectives include the regulation of malignancy-associated expression of growth factors by manipulatory glycosylation (Gabius et al. 2012). Many of the advances leading to our current knowledge have come from disease situations where glycoproteins are implicated and the glycans they carry are responsible for aberrant biological behaviour. It is therefore necessary to identify the normal glycosylation patterns in order to demonstrate their significance in disease. The sites of tissue presence of glycoproteins are a fundamental part of this programme and depend on histological approaches to address this issue. The importance of lectins and antibodies is crucial in this respect (Roth 2011) and is covered further in the section on lectins in this theme issue. As implicated under “Glycosylation of proteins” above, advances in carbohydrate and supramolecular chemistry contribute to make custom-made reagents available (Murphy et al. 2013; Percec et al. 2013; Zhang et al. 2015a, b; Roy et al. 2016; for a histochemical application, please see Roy et al. 2017, this issue). This is relevant for histochemistry and cell biology, where design and preparation of new molecules can support improved detection and specificity analysis.
